# School-based adolescent health survey in Himachal Pradesh, India: study design and protocol

**DOI:** 10.3389/fpubh.2024.1463776

**Published:** 2024-12-16

**Authors:** Saroj Mohanty, Devika Mehra, Subha Sankar Das, Gaurav Sethi, Rishi Garg, Anmol Gupta, Anjali Chauhan, Sunil Mehra

**Affiliations:** ^1^Mamta Health Institute for Mother and Child (HIMC), New Delhi, India; ^2^Medeon Science Park, Malmö, Sweden; ^3^Indira Gandhi Medical College, Shimla, India; ^4^National Health Mission, Himachal Pradesh, Shimla, India

**Keywords:** adolescent, India, school based, survey, Himachal Pradesh, study, research

## Abstract

**Background:**

The World Health Organization defined adolescent age group as a life span between 10 and 19 years. Adolescence is a phase of transition from childhood to adulthood, it is a unique stage for human development and a very sensitive period for laying the foundation of good health. Investment in this life-stage yields triple dividend. To promote health and well-being of adolescents, Government of India has launched various programs. An institutionalized evaluation of documenting achievement would strengthen these government led programs.

**Methods:**

A cross-sectional survey will be conducted among adolescents enrolled in government schools in the age group of 13–17 years. Two research instruments for students, principals, and for data collectors a checklist will be developed. Standard measures will be followed for data quality assurance. Data from students and principals will be collected in self-administration mode. Bivariate and multiple logistic regression will be employed for data analysis.

**Results:**

The study protocol for the school-based adolescent health survey will be implemented in Himachal Pradesh, India. The survey results will be disseminate both at state and national level.

**Conclusion:**

The study findings will provide policymakers and program planners with scientific evidence on key thematic areas essential for adolescent development and health outcomes. These insights will help identify and prioritize specific focus areas within the school health program, guiding more effective implementation strategies to address the unique needs of adolescents.

## Introduction

The adolescence phase between 10 and 19 years, is a very sensitive period for human development and transition from childhood to adulthood laying the foundation of future health. This formative phase affects adolescents regarding how they feel, think, make decisions, and interact with the world around them (1). Unmet health needs during adolescence, can affect their immediate growth and development, and have an impact on their future life course, with the potential of lasting effects on health of the next generations as well. However, investment in this life-stage can yield a triple dividend (2).

India has the largest adolescent population globally, with 253 million individuals in this age group. This means that every fifth person in India is between 10 and 19 years (3). Distribution of adolescents particularly in low- and middle-income countries like India, underscores the critical need for policies and programs that prioritize their well-being. These initiatives are essential to harness the full potential of this demographic, which plays a significant role in shaping the nation’s future. Fostering adolescent health not only promotes individual growth but also lays the groundwork for a sustainable and equitable future.

Acknowledging the vulnerability and significance of safeguarding and promoting the health and well-being of adolescents, the Government of India in January 2014, under the Ministry of Health and Family Welfare, launched *Rashtriya Kishor Swasthya Karyakram* (National Adolescent Health Program) (4). To achieve the Sustainable Development Goals 2030, both health and education are fundamental. Schools create a unique opportunity to improve the formal education and health education for adolescents. The Indian government initiated the *Ayushman Bharat* (Long life India)-School Health and Wellness Program (5) in 2018, in a joint collaboration between the Ministry of Health and Family Welfare and the Ministry of Human Resource and Development to strengthen the preventive and promotive health through activities for adolescents.

For decades, surveys such as the National Family Health Survey (6–10), Global Adult Tobacco Survey (11, 12), Global Youth Tobacco Survey (13–17), Global School Health Survey (18), National Mental Health Survey (19), and Comprehensive National Nutrition Survey (20) have provided critical insights into India’s public health landscape. However, there remains a significant gap in the adolescent health domain, with no comprehensive survey dedicated to evaluating adolescent health programs. However, no comprehensive survey has been conducted to specifically evaluate adolescent health programs. A structured, institutional evaluation focused on documenting achievements, identifying challenges, and assessing outcomes in adolescent health would significantly enhance government-led efforts. Such an evaluation would provide a deeper understanding of program effectiveness, inform future policy strategies, and ensure that adolescent health initiatives are aligned with the specific and evolving needs of this population.

Adolescents (15–19 years) in Himachal remain vulnerable, according to the National Family Health Survey-5 (10), as 53.2% girls and 22.1% boys were anemic, 4.9% adolescent girls and 6.4% boys were overweight/obese, and 3.4% girls were either already mothers or pregnant. The survey further reported that 33.2% girls and 28.4% boys, use modern methods of contraception. Substance use, particularly tobacco use, is also significant with 14% tobacco users (aged 20–34 years) having initiated tobacco use at the age of 15–17 years (12).

The overall aim of the study is to comprehensively assess adolescent health-related issues, behaviors, and service utilization among school students using the Global School-based Health Survey framework. The specific objectives of the Adolescent health survey would be to:

1. Assess the prevalence and types of adolescent health-related issues, including health behaviors, to establish a comprehensive understanding of current health challenges.
2. Evaluate access to and utilization of services and commodities made available to adolescents under the *Ayushman Bharat* School Health Program and *Rashtriya Kishor Swasthya Karyakram*, identifying any service gaps.
3. Examine the policy environment and practices in schools related to adolescent health promotion, providing insight into the effectiveness of existing strategies and policies.
4. Suggest recommendations to enhance and strengthen the ongoing adolescent health programs based on findings, aiming for impactful and sustainable improvements in adolescent health.

The survey results would empower the State government to design, need based interventions and programs or enhance existing initiatives, allowing for a more effective response to adolescent health needs. These insights would help tailor approaches to ensure they are relevant, impactful, and aligned with the unique needs of the adolescent population, ultimately fostering improved health outcomes for this formative age group.

## Methods and analysis

### Study setting

Himachal Pradesh a state in India is divided into 12 districts and further divided into 73 sub-divisions, 78 blocks, and 172 tehsils. According to the Census, 2011 the literacy rate of the state was 83.78% which was well above the national average (21). In the State, 928 government high schools, 1869 government senior secondary schools, and 130 government colleges are enrolling students. Meeting the constitutional obligation to make primary education compulsory, the state became the first state in India to make elementary education accessible to every child (22). There is almost universal enrolment of students as the Net Enrolment Ratio was 99.7 at the primary level and the retention rate was 96.42 in class 11th (23). Himachal Pradesh is an exception to the nationwide gender bias in educational access (24). School enrolment and participation rates for girls are almost universal at the primary level. While higher levels of education do reflect gender disparity, Himachal Pradesh is still significantly ahead of other states in bridging the gap (25).

### Consultation stage

Before initiating the study, two advisory committees will be constituted to guide the survey, one at the National level comprising of experts in adolescent health and research methodology. The second one at the State level which will include government officials from health, education, economics, statistics departments and academia. These committees will advise on study design, survey tool development, sampling methodology, survey planning, data quality assurance, data analysis, and finalizing the study report. Bimonthly meetings will be held to ensure guidance throughout the study.

### Study design and participants

A cross-sectional design will be employed for this study. For Objective 1, the participants will be adolescents, while Objectives 2 and 3 will include both adolescents and school principals as participants. Objective 4 will be addressed following the survey results by integrating findings from a literature review. The inclusion criteria that will determine student participation in the survey would be (i) students within the age group of 13–17 years who are present on the day of data collection, (ii) parents of students who would give passive consent for their children to participate, and (iii) students who would give assent to participate in the study. The exclusion criteria would include: (i) Parents of students who will not give passive consent for their children to participate (ii). Students who will not give assent to participate in the survey (iii) Students who will be absent on the day of data collection.

Permission from the State government for this survey could be a challenge, especially given the need to coordinate with multiple departments, including Health and Family Welfare and Education Department. Thus, we will engage with the heads of these departments early in the process, clearly articulating the survey’s importance, anticipated benefits for adolescent health, and the positive impact on the State’s future.

### Sampling

The total number of students (age 13–17 years) enrolled from class 8 to class 12 is 7,82,941 (23) in the academic year 2020. District-wise school enrolment data containing school type (secondary, higher secondary), enrolment type (exclusive boys, exclusive girls, co-educational), location of the school (rural, urban), name of the school, name of the school cluster, the total number of students enrolled in each class, sex (boy, girl) of the enrolled student, will be collected from the education department which will serve as the sample frame.

Cochran sampling technique of finite population (26) will be used to determine Sample size for the study as:

$\begin{array}{l} n'=[{\left(M\right)}^{*}\left(N^{*}\left(z^{2}\right)*p^{*}\left(1-p\right)\right)/\\ \;\left({\left(d^{2}\right)}^{*}\left(N-1\right)+\left(z^{2}\right)*p\left(1-p\right)\right)] \end{array}$

Where, n' = Sample size, M = Number of sampling stages, N = Universe/total population, z = Confidence level (1.96) for 95%, p = Prevalence (0.05), and d = Margin of error (0.5).

In addition, 15% sample of the respondents will be added to account for non-response and/or incomplete responses. The sample will be calculated for the State initially, followed by distribution of sample across 12 districts using the Probability-proportional-to-size. Location of schools (rural and urban), sex of the respondents (boy and girl), and age of adolescents (13–15 years and 16–17 years) will be used. Exclusive boy’s or exclusive girl’s schools (which are not coeducational) will be considered for sample distribution at the first instance. It will be ensured that one school from each school cluster (a school cluster in India is the grouping of schools that are geographically close together to share educational resources and instructional materials with the purpose of improving educational standards) is included for sample distribution.

Obtaining school wise enrolment data for districts from the Education department is another potential hurdle, as such information is often deemed confidential. To address this, emphasizing the survey’s alignment with the anticipated benefits for school-enrolled students can be instrumental. By highlighting value of the survey in enhancing educational and health outcomes for students, and maintaining an institutional guarantee of data confidentiality and restricted use strictly for survey purposes, the department may be more inclined to consider sharing the data. Further the State advisory committee would also be instrumental for inter departmental coordination.

### Measurements

Three research instruments will be developed (2 self-administered, and 1 observational checklist). The self-administered instruments will be for the students, and principals, while observing the school environment the checklist would be used. The inclusion of both adolescent respondents and school principals will provide a comprehensive complementary understanding of adolescent health issues and school-level factors impacting health outcomes. This multi-layered approach from adolescents, school principals, and direct observation will complement the findings and enhance the understanding of factors influencing adolescent health.

1. Student survey (self-administered) will be adapted from the ‘Global School-based Student Health Survey’ (18) based on the state context covering the following domains: dietary behavior, physical activity, hygiene, digital devise use and cyber bulling, violence and road safety, connect to parents/guardians, health seeking behavior, access to government schemes, substance use, reproductive and sexual health, menstrual hygiene, and mental health. The survey tool will consist entirely of close-ended questions, with lesser than 100 questions in total. Each question will offer, on average, three response options.
2. Principal survey (self-administered) will use the ‘Global School Health Policies and Practice Survey’ (27) covering the thematic areas of school health coordination, healthy and safe school environment, violence and bullying, substance use prevention, implementation of government schemes, and physical education to understand the school policy environment and practices promoting health and wellbeing of adolescents in schools.
3. Observation checklist will be developed with the aim of capturing the school environment on issues such as physical cleanliness, substance use, availability of vendors selling junk food, adherence to Tobacco Free Education Institution guidelines and school attendance as observed on the day of the survey. Below mentioned framework will be used in the survey (Table 1).

**Table 1:** Framework of modules for data collection.

Modules for adolescents	Modules for principals	Checklist for data collectors
Food Preferences and Choices	Healthy and Safe School Environment	Shop(s) selling tobacco products within 100 yards
Physical Activity	Violence and Bullying Prevention	Display of “Tobacco Free Education Institution” signage
Hygiene	Substance Use Prevention	Cleanliness of School
Digital Device Use	Implementation of Government Schemes	Handwashing Facility
Connect to Parents and/or Guardian	Physical Education and Activity	Running Water Facility
Health Seeking Behavior		Cleanliness of Toilets
Access to Government Schemes		Cleanliness of dustbins
Violence and Road Safety		
Substance Use		
Reproductive and Sexual Health		
Menstrual Hygiene		
Mental Health		

Adoption of the survey tools by the state government may indeed be challenging due to sensitive questions in areas such as personal hygiene, violence, substance use, reproductive and sexual health, and mental health. The data collection will be entirely anonymized, with no identifiers, and conducted via a self-administered approach to ensure privacy. The draft questionnaires would be veted by the State Advisory committee further. Additionally, the State consultative process will focus on the relevance of these domains to the successful planning and implementation of school-based programs will be highlighted, illustrating how these insights are vital for improving adolescent health, well-being, and empowerment within current environment.

KoBo collect (28) will be used to create these survey tools electronically.

All tools will be pilot-tested for content validity and face validity. Content validity will be established by seeking inputs from the domain experts. Pilot testing will be conducted to assess the face validity in two districts, with representation of schools other than the sampled schools from rural and urban areas, and will include adolescent boys and girls. Feedback from experts and adolescents related to the language of the questions, sequencing, topic sensitivity, and addition or deletion will be incorporated into the survey instrument to enhance its appropriateness before commencing the data collection. Face-to-face interviews will be conducted for assessing group concurrent validity among 5% of the respondents, along with data collection in self-administration mode and the results will be compared before actual data collection. If warranted, survey tools will be titrated.

Pilot testing may pose challenges such as delays in obtaining passive consent from parents, non-cooperation from school authorities, or unexpected school closures. To mitigate these, schools will be informed by the concerned State and Block authorities well in advance about the pilot, and ample consent forms will be distributed ahead of time to ensure parents have sufficient time to review. School authorities will also be briefed on the importance of the survey for adolescent health, which can help secure their cooperation. For any unplanned school closures, backup planning for rescheduling will be in place to minimize disruptions to the timeline.

### Data collection

A two-day interactive residential training session will be organized in the state capital Shimla, in which an estimated 24 data collectors (2 per district) having post-graduation qualification and having experience in data collection will be trained on the survey tools, field operational plan, data quality assurance, and anticipated challenges and probable solutions. On the subsequent day, they will practice using the research instruments in non-sampled schools. The non-sampled schools will be matched as those of sampled schools like location (rural and urban), category (high school, higher secondary school), and type (coeducational, exclusive boys, exclusive girls). So, data collectors will be exposed to both classroom and practical based training. They will share their observations and discuss regarding the problems faced and probable solutions. Additionally, the data collectors would also be running dummy testing in schools, so that problem noted can be addressed and based on these, the field operational plan will be titrated.

The research tools will be translated into Hindi (local language of the state) first and followed by back translation by an independent team into English to ensure that the meaning of each question is not diluted. Data will be collected from adolescent respondents and school principals. The collected data will be uploaded in real time or wherever the internet connection will be available. Additionally, the data collection team will use the observation checklist while on respective school premises and the surroundings as per the prescribed format.

Data collection across all 12 districts may pose various seasonal variations challenges, difficult terrain, diverse school schedules, and potential resistance from school authorities. To address this, a carefully designed field operation plan will ensure that data collection concludes before adverse weather conditions and aligns with local educational calendars. Local data collectors familiar with the terrain will be hired to enhance navigation and efficiency. A joint communication from the Education and Health and Family Welfare departments will be issued to school authorities, emphasizing the survey’s prevalence and easing any concerns.

### Ethical considerations

Parents or legal guardians of the adolescents will give their passive consent (29) (parents/guardians to sign and return the consent form if they refuse to allow their child to participate in the study). Electronic student assent will be taken after obtaining consent from respective school authority. Students who would not give the assent will be excluded from the data collection process. Students would be explained that the participation in the survey is voluntary and confidential. Similarly, respondents can withdraw from the survey at any point of time if they feel uncomfortable answering any question and a respondent will also have the right not to respond to any particular question(s). Ethical approval for the study will be obtained from the Institutional Review Board of Mamta Health Institute for Mother and Child, New Delhi.

Securing approval for parental passive consent from the Institutional Review Board could be challenging, given the preference for written consent in India. To address this, we will present evidence supporting passive consent practices used globally, showcasing its effectiveness and ethical acceptance in similar contexts.

### Data quality assurance

Logical sequencing, question skip pattern, default global position system locations, and the date and time of data gathering will be maintained in the data collection interface. For the identification of student respondents in each school, an automated calculating mechanism will also be integrated into the interface.

Incomplete or missing data could reduce the number of observations for the survey outcome. A rigorous quality assessment framework will be developed and strictly followed. Key aspects of this framework will include: double-check and validate the data collection in individual schools. Contact details including phone numbers of the principals of the sampled schools will be collected. For 25% of the selected schools, a data quality team with experience in ensuring data quality parameters will accompany the data collectors during the data collection process. Back checks and spot checks will also be undertaken by this team. Using the KoBo collect, data will be analyzed daily to identify outliers, unusual patterns, refusal rates, responses of key questions, and deviations observed. Throughout the data collection process, at a scheduled time every evening, data collectors and the study management team will be connected online to discuss challenges, based on findings of the quality management team to decide the future course of action.

### Data analysis

The collected data will be cleaned and edited for inconsistencies. Descriptive statistics across variables will be used. For analytical purposes, variables will be categorized into dependent and independent variables, and transformations will be applied as needed to convert variables into categorical forms. A bivariate analysis using the Chi-square test will be conducted to examine associations between each independent variable and the dependent variable, with a significance threshold set at *p* < 0.05. Following this, a multivariate logistic regression analysis will be performed with the dependent variable, incorporating all independent variables as predictors. The results will be presented as adjusted odds ratios, providing insights into the likelihood associated with each independent variable. Data analysis for the study will be conducted using IBM SPSS Statistics 22 (30) or Stata 18 (31).

To address the anticipated challenges of missing values and responses categorized as “Others” when specific options are available, 15% additional Sampling will be considered which will help offset any data loss from missing values.

### Study duration

The study duration will be of two and half years starting from day of execution.

## Discussion

Precise estimation of the prevalence of adolescent health issues is critical for service planning and prioritization, as well as for advocacy and public health campaigns targeted at increasing awareness. In Low- and Middle-Income Countries including India, the systematic and longitudinal recurrent measurement of adolescent health and well-being remains particularly, uncommon. This gap is notable due to several shortcomings, including a lack of coverage for adolescents, diverse ethnicities, and religions in existing large scale surveys in India. Age and sex disaggregated data are often lacking, partly due to incomplete age coverage, limiting its critical use for program planning. Additionally, there is inadequate coverage of domains such as mental health, substance use, injury, sexual and reproductive health among unmarried adolescents, and positive aspects of adolescent health and well-being. Current adolescent health data systems often lack inter-sectoral coordination beyond health, such as with education, water and sanitation, and social protection systems (32).

The methodology adopted in this study has the potential to be scaled up at the national level, as it will represent the first-ever state-level school-based adolescent survey experience in India. This survey will be beneficial for the government and adolescents due to its comprehensive approach. This methodology can also guide future adolescent health surveys, providing a standardized framework for data collection and analysis.

The study outcomes will aid the Global Action for Measurement of Adolescent Health initiative which promotes harmonized guidance for adolescent health measurement, supporting countries and technical organizations in collecting useful data to track progress in adolescent health (33).

This study will offer a valuable opportunity to strengthen the ongoing adolescent health programs by addressing the identified gaps in Himachal Pradesh for health and wellbeing of adolescents.

## Strength and limitation

The strength of the study is that this will be the first adolescent health survey to be conducted at the state level in India with district- level sample representation. To the best of our knowledge, no previous survey has employed such rigorous methodology specifically focusing on adolescents—a population often overlooked in health surveys. One of the limitations of this study is that due to the cross-sectional nature of the survey we would not be able to establish causality of the associations. Additionally, out-of-school adolescents and chronically absent students will be beyond the scope of this study, which may limit the representativeness of the findings.

## Conclusion

This protocol paper outlines methods for developing adaptable tools that can be used across diverse populations to gather comprehensive data, thereby addressing gaps in adolescent health research. Additionally, the study findings will equip policymakers with need-based insights into adolescent health themes that need to be emphasized within the existing school health program.
